# Management succession and success in a professional soccer team

**DOI:** 10.1371/journal.pone.0212634

**Published:** 2019-03-13

**Authors:** Paul Kattuman, Christoph Loch, Charlotte Kurchian

**Affiliations:** Cambridge Judge Business School, University of Cambridge, Cambridge, United Kingdom; Queen Mary University of London, UNITED KINGDOM

## Abstract

Research into sports team performance has shown that across many sports and league competitions, teams that change their coaches after a decline in performance do rebound, but fare no better on average than teams that have not changed their coach in a similar situation. A similar lack of succession benefits has been reported in studies of manager and CEO succession: it has not been established that changing a team’s leader improves a declining team’s performance. We study the effect of a change of coach on the performance of a professional soccer team. Based on rarely obtained access to a whole season (one year) of daily close observation of the team and coaching staff in practice and matches, this study uses quantitative and qualitative data to go beyond the “average” pattern reported in the literature. We document in detail how, in a single team case study over an entire season, the *processes* in leadership behavior changed with a change of coach, the effect this had on the state of mind of the team, how the match behaviors of the players changed, and how these changes translated into improved performance. The process effects of a leadership change on the performance of a sports team may hold insights for leader succession in management: in addition to the aggregate organizational and experience fit of the new team leader, the specific leadership processes introduced by the new leader are critical for performance effects.

## Introduction

How important is a change in leadership for the performance of a team? The ‘conventional wisdom’ is, not at all: although the performances of soccer teams tend to improve on average after a change of coach, the improvement is not driven by the change in leadership; rather, ‘recovery’ from a poor performance streak happens regardless of whether the coach is changed or not [1–9]. This ‘regression to the mean’ argument has entered popular folklore [10–11].

The “no-effect” conclusion is based on studies that have averaged the experiences of multiple teams. An alternative interpretation is that a team-leader change makes a difference, for better or worse: some new leaders improve performance while others worsen it; these on average cancel each other out. This account, if true, highlights the importance of understanding how precisely coach behaviors affect team performance. To examine this view and its implications, one needs to focus on single teams first, characterizing in detail the behavioral changes that accompanied the coach change and any subsequent performance changes to generate hypotheses about underlying processes. Only then, a study of multiple teams can test these hypotheses in ways that avoid the masking effect of ‘averaging’.

This study undertakes the first of these two steps: we analyze evidence gathered through detailed observation of a single professional soccer team, with one of the authors at the training ground and matches through an entire season (one year), observing in detail the behaviors of the coach and team members. Fortuitously, a change in coach occurred in the middle of the season after a run of deteriorating performance. This ‘natural experiment’ enabled us to examine the behavior difference between the new coach and the old, its association with the change in behaviors of the players and the team as a whole, and how this was associated with a change in performance.

The leadership challenges in sports teams have parallels with those in wider organizations [12–13]. The management literature also contains similar ‘leader change irrelevance’ results. For example: ‘The effectiveness of replacing managers is doubtful at best. For example, most companies perform no better after they dismiss their CEOs than they did in the years leading up to the dismissals’ ([14], p. 70). Of course, CEOs are responsible for entire organizations rather than teams. However, CEOs do run teams of senior managers, and they have often been compared to soccer managers in terms of motivation responsibilities, e.g., [9]. Our study of sports leader succession may offer insights for leadership succession in organizational teams.

## Review of previous work and theory development

### The seeming irrelevance of leadership succession

Sports team research has largely concluded that, while the performances of soccer teams appear to improve after a change of manager, this is not a causal effect. [1] compared the average performances in the Dutch league over four games before and after a change of coach, with the average performance of a control group with four losing games in a row but no change in coach. Both groups rebound after a losing streak, and the rebound pattern cannot be clearly distinguished between those with and those without a change of coach. Similar results have been reported in [2], and the English [4], French [15] and Colombian leagues [7]. Multiple other studies find consistent results [3, 5, 8, 9]. Only one study, examining the English premiership, suggests that changing the coach has a short-term positive effect but is damaging in the longer run [16]. Another study finds a positive effect of coach change but fails to compare teams that changed the coach with teams in similar situations that did not change the coach [17].

Several studies have attempted to determine the effects of explanatory variables on leader performance. [18] found that, in the English Premier League, bringing in a new coach after the old coach leaves in an orderly manner is better for performance; and bringing in a coach with international experience is better than bringing in one with domestic experience. [9] concluded explicitly that in the face of the irrelevance of coach changes, the high number of coach sackings suggests that coaches are indeed scapegoats.

There is also a strand in the sports management literature of predictive data models that use player salaries, transfer investments, number of players available, or number of games beyond the core league (e.g., in European competitions), to statistically predict performance and isolate the residual as a reflection of coach skills [19–21]. None of these studies, however, examine managerial and team process variables directly in order to explain how exactly what a coach does might influence performance.

In the literature on teams and organizations too, the effect of leader succession is inconclusive. Since the 1950s [22–24], conflicting theories and evidence using a variety of approaches have accumulated. Succession effects have been found to be positive [25], negative [26] and irrelevant [27].

Three groups of theories of leadership succession have provided frameworks to understand some of the apparent discrepancies [28]. The ‘common sense’ theory [29] posited that a good new leader could bring fresh perspectives to the organization, resulting in a positive performance effect. An opposing ‘vicious cycle’ theory [29] argued that succession might disrupt routines, exacerbating the existing performance problem. In this vein, military leader succession has been argued to lead to a deterioration in performance, based on data of US army deployment in the Korean war [30].

The ‘scapegoat theory’ contended that there was no relationship between senior leader succession and the organization’s performance. Thus [31] claimed that Grusky’s data did not offer evidence of causality; rather, the fired team managers were ‘scapegoats’–team effectiveness was driven more by recruiting policies than succession.

The effects of leadership changes at the top of organizations are equally unclear. New CEOs might achieve better returns than their predecessors [32], but such improvements may be ‘bought’ by lowering investments in R&D and pension funds [33]. The hiring of external CEOs tends to be less successful than the hiring of insiders [34–35]. Indeed, [36] concluded: ‘We are no closer to finding a general theory for (…) the impact of leader succession on performance’ (p. 981).

The motivation for this study is our view that inconclusive findings on the effect of coach changes cannot be interpreted as meaning that these changes do not matter. Prescriptions about how to select a new coach can only be developed based on process studies that examine the dynamics of how a new leader acts to change performance.

### Team processes and the importance of leadership

[37] defined leadership as ‘the behavioral process of influencing individuals and groups towards set goals’. There is substantial evidence that team leaders do enhance social processes of problem-solving and team processes [38], and they can help teams to learn [39]. *Trust* in leadership has been found to enhance sports performance [40]. If the leader matters, then so does a change of leader. The organization should invest care in leader selection: ‘A good board can make a CEO replacement pay off if its members first develop a better understanding of the business context, worry less about pleasing the investment community and more about a replacement's strategic fit’ ([14], p. 70).

Motivational states are important factors in team performance, supporting, for example, the management of self, team efficacy and anxiety control [41]. [42] found that positive feedback from a coach helps to maintain a positive affective (emotional) state of the group, which can improve performance. ‘Leaders who feel excited, enthusiastic, and energetic themselves are likely to similarly energize their followers, as are leaders who feel distressed and hostile likely to negatively activate their followers’ ([43], p. 84, supported by an experimental study [44]).

The influence on team processes and group affect by a leader are observable. In turn, group affective states can influence performance outcomes through, for example, anxiety control, cooperative behaviors and risk-taking [45], which are also observable. Positive states can influence efficacy judgments, whereas negative states can encourage thorough problem-solving [46].

### Theory and propositions

In order to understand whether, and how, a leadership change affects team performance, we need to look in detail at what is going on inside the team rather than focus on aggregate patterns in the more readily observable secondary data, averaged across multiple teams. The insights on the effects of a specific leader’s succession on leadership actions, team processes and performance gained thereby can be extended to the analysis to multiple teams, to understand whether leaders’ actions have consistent effects on performance across teams.

This study performs the first of these two steps: focusing on a single team, it examines detailed evidence on differences in behavior between two coaches, and the associated changes in the team’s internal dynamics and performance. The second step, comparison across many teams, is beyond the scope of the current study.

**Fig 1:**
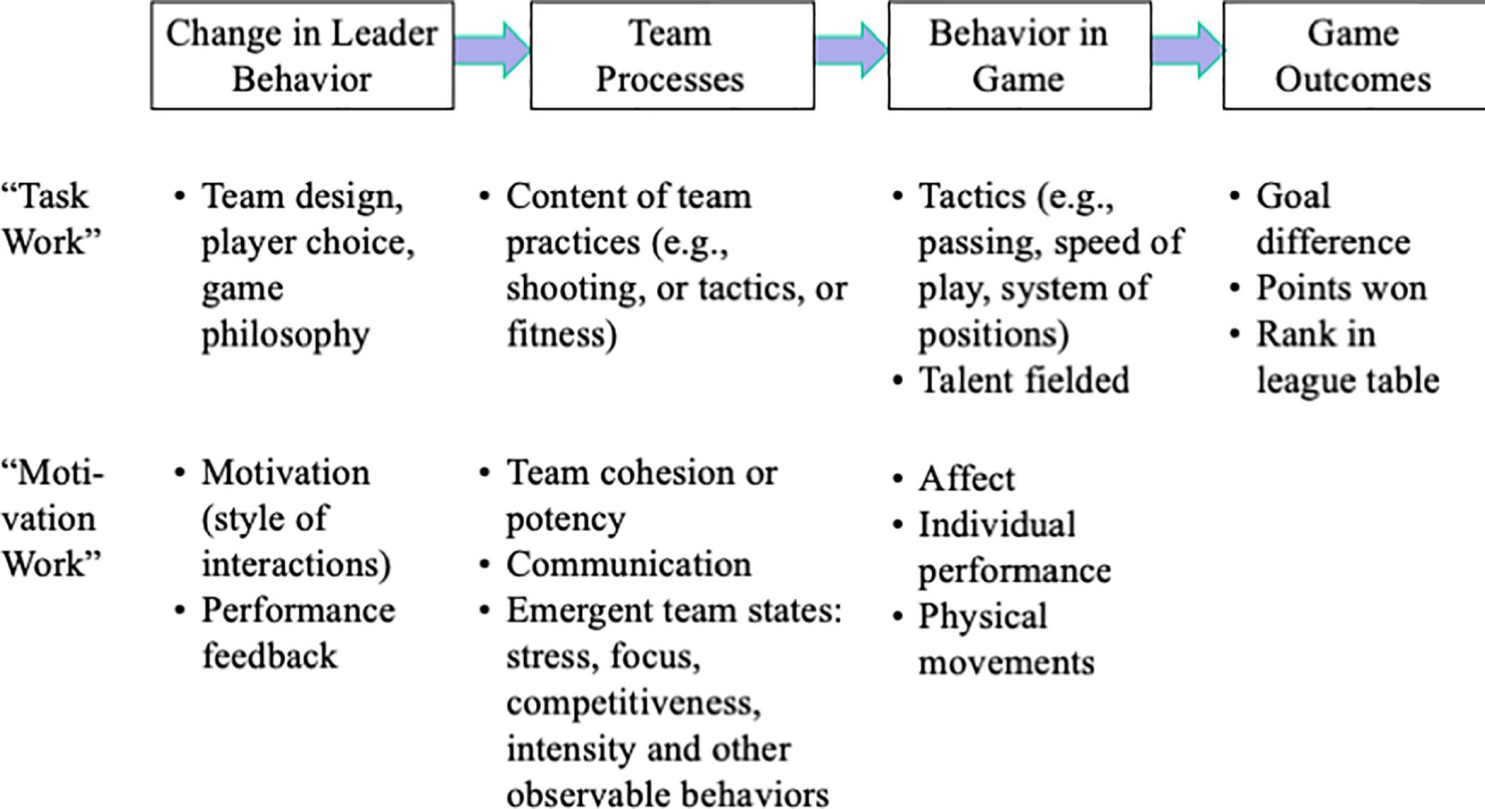
Effects associated with change of leader.

We summarize the discussion of theory in a conceptual framework (Fig 1, based on [38]). As input, the leader chooses the team (talent on the field), sets the game strategy and approach, and drives motivation through his/her leadership and feedback style. These inputs drive the team processes: on the task side, physical aspects of the practice sessions leading up to the match; and on the motivation side, psychological aspects such as team cohesion and emergent team states. For the match, the coach sets a tactical plan. The inputs and processes together influence the affect and effort levels, which in turn influence game behavior and team performance. Performance feeds back to the drivers for the next performance situation.

We exploited the research opportunity provided by a change in the host team’s coach in the middle of the season. This enabled us to observe the effects of leadership succession closely for this team. We investigated whether the behavior of the new coach had an effect on the team processes and game behaviors, structured by the following propositions.

First, leadership research suggests that the leader can influence the team and, consequently, its performance. Leaders contribute by choosing players and motivating them, setting game plans and influencing the emotional dimensions of teamwork [47]. These factors have the potential to change performance-relevant ways in which the team employs tactics on the pitch, and how it handles emotional events such as conceding a goal. Thus, overall:

**P0:** *Changing the leader is associated with a change in team performance*.

We articulate the next three propositions conditioned on improved performance at three levels. First, prior research suggests that improved performance results from positive leader behavior. When leaders can unite a team behind values, group effectiveness improves [48].

**P1.** *An improvement in performance is associated with more positive leader (motivating and feedback) behaviors by the new coach*.

Teams develop shared attitudes and behaviors through shared experiences [49]. Emergent states are ‘cognitive, motivational, and affective states of teams [that are] dynamic in nature, and vary as a function of team context, inputs, processes, and outcomes’ ([50], p. 357). Emergent states are predictors of performance [51], influencing shared problem-solving and cooperation.

**P2.** *An improvement in performance is associated with a positive change in the emergent states of the team (e*.*g*., *stress*, *cohesion*, *intensity or focus)*.

Game performance is determined by players’ behaviors during the game. Soccer is a complex strategy game requiring a wide range of behaviors: effort (e.g., distance run, number of sprints), focus (e.g., tactical discipline, capability to execute technical skills), collaboration (e.g., unselfish passes, backing one another up, mutual encouragement), and positive affect and self-confidence (enabling, e.g., maintenance of competitive effort after falling behind, and efficacy of actions).

**P3.** *Improved performance is associated with positive changes in game behavior (individual performances of players)*. *This is a combination of* **P3a**: *tactical game behavior (such as passing patterns and running patterns) and* **P3b**: *the affective states of the players during the game*.

## Method and data

As stated in the introduction, the premise of this study is that deep observation is necessary to identify team-level performance effects, because averaging over the experiences of multiple teams can lead to a fallacious "no-effect" result. This requires detailed daily observation of coach and team behavior and performance over a sufficiently long period of time. However, such access is rarely granted by soccer clubs due to their sensitivity to external observation. The choice of the team to study was necessarily governed by the prospect of obtaining access. Thus, this paper reports on a case study of one European professional soccer team with close observation over an entire year, to generate hypotheses about coach behavior performance effects. Access was granted with some constraints: only one of the authors could be present, as a passive observer; player interviews, questionnaires and interaction in any other way were prohibited, which excluded a fully ethnographic approach. The CEO of the club provided written, informed consent to the study; no individual data of any of the subjects was used. The study was approved by the departmental ethics review group of Cambridge Judge Business School, approval reference 16–029.

The authors double-coded emerging variables during the six weeks of pre-season, generated categories, and calibrated and finalized the set of variables to be collected during the season (following [52]). Direct observation (*in vivo* coding) has the advantages of objectivity and recall over retroactive reports by team members and intrusive physiological measurements [53]. We attempted to benefit from the advantages of the imposed data-collection method while overcoming its limitations.

Formal data collection began on the first day of the season. The CEO of the club and the coach introduced one of the authors as a ‘performance psychologist’, who would assess whether the team’s environment was conducive to performance. No evaluation of any individual player would be undertaken. The researcher proceeded to observe four-hour-long training sessions, four days a week, and weekly matches over the following ten months. Close observation from the sidelines during training and down time followed the protocol of ‘patrolling’ the field and lunch/break room, to catch as many interactions as possible. Changing rooms were out of bounds, but the observed interactions represented a substantial and representative sample. Observations were recorded using well-defined, easy-to-record count variables, supplemented with nuanced details such as the tone of voice and body language of both coaches and players, and tallied every day. We also captured quotes from coaching staff and players, enabling a more detailed interpretation of the statistical results. The data was collected in four categories, consistent with our theory framework: coaching behavior, team processes and emergent team states, game behavior, and game outcomes.

At every step, we guarded against researcher bias. One measure we adopted was to rely on well-defined and easy-to-record count measures for our variables. Further, the variables in our analysis were predefined based on the six weeks of pre-season observation. Especially the numerical team affect scores during games were compared with evaluatory write-ups of a club representative. The high correlation between them assured us of the robustness of our measurements.

Nearly midway through the season, in week 20, the coach was replaced. This provided us with the opportunity to examine differences in coaching styles and their possible succession effects on team performance.

### Coaching behavior

The behavior of the coaches was measured using frequency counts of their interactions using categories that emerged from pre-season observation, shown in Table 1 with a note pertaining to each. (Descriptive statistics are presented in S1 Appendix.)

**Table 1:** Descriptions of coaching interaction categories.

Behavior Category	Description
1. Mild Praise	Small verbal or physical encouragement, such as *‘good job’* in a calm tone or quiet applause.
2. Strong Praise	Loud and excited verbal or physical encouragement or celebration, such as *‘Brilliant*!! *Amazing*! *Best yet*!*’* or loud enthusiastic applause.
3. Mild Support	Some type of care, such as asking if the player is doing okay or feeling well.
4. Strong Support	A more emotionally involved type of care, such as an arm over the shoulder in support of a poor performance or a personal long talk.
5. Mild Friendly	A simple interaction, such as a happy *‘Good morning’* or a pat on the back.
6. Strong Friendly	An enthusiastic interaction with no purpose other than being friendly, such as joking loudly together or other very positive verbal and physical interaction.
7. Mild Criticism	Something negative, but which does not seem very emotional or where the tone is not particularly strong, such as *‘You think you're something that you're not*.*’*
8. Strong Criticism	An emotional, negative verbal or physical interaction, such as *‘You are hopeless*!*’*

The categories reproduce previously proposed leadership behaviors: the coach provided performance feedback, directly through ‘criticism’ (items 9 and 10) and ‘praise’ (items 1 and 2), and combined with instructions on what to do better in ‘challenges’ (items 7 and 8). The coach also simply provided encouragement and ‘support’ (items 3–6).

### Emergent team states

Emergent team states are the dynamically changing cognitive, motivational and affective states of teams [50]. They include ‘group affective tone’ (‘consistent affective reactions within a group’ ([43], p. 78). We rated the players’ *collective* motivation, positivism, self-confidence and other observable cognitive or emotional states that were potentially performance-relevant (Table 2).

**Table 2:** Descriptions of team affective tone variables.

Dimension	Description
Relaxed	A sense of ease, contented feelings and a steadiness of the emotions of the group. It doesn’t have to be calm, although it can be. The higher the score, the less the feelings of unease, stress or other negative emotions and motivations.
Laughing	Extent of joking, laughing and smiling. This is separate to being relaxed. You can be somewhat relaxed but not in a jokey mood. It indicates a different kind of energy than just calmness or some excitement or closeness in the group. The lower the score, the more serious the players seem.
Tension	The emotional arousal creating a readiness for action by preparing muscles and focusing the mind. It can include an edge of nervousness. If it’s strongly negative and emotional, it turns into stress, but if it prepares the players for work, we classify it as tension. The rating is determined by the body language and speech of both players and coaches.
Stress	A negative, upsetting, unsettling set of events and emotions. It is based on the atmosphere or on significant events that color the day, such as the team being reprimanded. It could also be the remnants of a loss or a team failing that bring down the atmosphere. It is different from tension, which may be positive, negative or neutral, while stress here is always negative. A high score indicates an overwhelmingly negative day with many negative experiences and everyone feeling stressed.
Aggression	The energy the players put into tackling one another in training, play fighting, real fighting and swearing, and how much energy they put into their physical attack and defense. It can be positive or negative, as these can become blurred. A high score denotes a lot of physical action during training, whereas a low score indicates very little.
Competition	The extent of how much they want to win the exercises and how much they are competing socially–observable by the amount of energy expended, and the extent of their disappointment or celebration after exercise outcomes. It is related to aggression, as they tend to get more aggressive when they really want to win, but it’s not aggression for its own sake. A high score means they were very focused on winning the entire session and there was probably something at stake, such as a prize or a place on the team.
Cohesion	The players’ relationships and how much they express their closeness. A high score indicates that they seem close as a group and there have been some notable group identity moments that day, such as all walking together after training, or a noticeable amount of fluidity across groups of friends.
Intensity of Session	A measure of the energy, emotions and tensions. It captures how much they were pushed by the coaches and one another, how much pressure they were feeling, and how excited and energetic they were. A high score indicates they were pushed very hard that session and responded well to that pressure.
Focus	The players’ concentration. A high score indicates no distraction and a willingness and activeness in the listening, learning and trying to perform well.

In categorizing pre-season observations, we adapted [54] recording approach (used in a study of teenagers) to the behavior of players and coaches. Nine stable behavior variables emerged. Each was rated (pre-season) on a scale of 1 to 10, along with descriptions. Several variables that emerged are consistent with the literature, such as cohesion [55], which is also close to team potency [56]. Descriptive statistics and correlations among these measures are shown in S1 Appendix.

### Game behaviors

We measured physical movements during the game, such as distance run and sprints, which are indications of effort. Second, we counted the passing frequencies between pairs of players during each game, as well as the passing accuracy (%). The passing network offers a representation of the game tactics (e.g., long balls versus short, or playing via the wings or through the middle).

Third, we obtained ratings by an internal expert (among the coaching staff) of each player’s ‘talent’. A 10 indicated the assessment that this player was ‘one of the few best players on this position in this league during this season’, a 5 indicated an ‘average player’, and a 1 ‘one of the few weakest players’. This rating represented a ‘team talent’ variable for each game, or the average skill of the players fielded in this game.

On the motivation side, we observed the team’s positive and negative *affects*. These are multidimensional constructs: of enthusiasm, energy and alertness in the case of positive affect; and distress and unpleasurable engagement in the case of negative affect. Various scales of positive and negative affects exist, and there is debate about whether positive and negative affects lie at opposite ends of the same scale [57–58], or whether they are independent [59]. We estimated positive and negative affects through careful observation of the players’ body language, and exchanges among them during each match, tallying counts of behavior and supplementing the counts with descriptions. Positive affect was detected in, for example, confident body language (head held high); physical displays of celebration; composure under pressure (calm handling of a tackle); speed of recovery after a negative event such as conceding a goal; positive interactions on the pitch (hi-fives, hugs, team huddles, supportive words); and dealing with aggression from the opposing team with composure.

Negative affect was indicated by postures of defeat (dropped heads); a lack of celebration after positive events; nervous actions under pressure resulting in errors (a clumsy pass or loss of ball in a dribble); a long time taken to recover from a negative event; a lack of positive interactions between teammates; negative interactions (arguing or fighting between team members); and instances of aggressive displays from the opposition resulting in detrimental behavior (such as a fight).

Negative and positive emotional events co-existed in the same match. We measured them separately, each in its own scale from 0 to 10. A 10 for positive or negative affect indicated that the above events of positivity or negativity were demonstrated regularly throughout the match. A 5 for either suggested that some were evident, and a 0 indicated a total lack of observable emotional behaviors. Thus, lower scores suggested that the match was less emotionally charged.

To check robustness, we obtained a reliability estimate by comparing our notes and scores with the post-match write-ups of a club representative who wrote evaluations of every match for the club’s social media. Comparing the tallies of both positive and negative emotionally charged language in the match write-ups with our affect scores for the match, we found a high correlation, supporting the validity of our measures.

### Game outcomes

The key game outcome in professional soccer league competition is the game result: win, draw or loss. However, this categorical variable filters out useful information. It has been argued that *goal difference* is a more informative statistical measure of game outcome [60]. The primary variable that we use is the *league table rank*–this settles promotion or relegation at the end of the season and is the main variable driving the behavior of coaches and the CEO. This variable is most commonly used in studies that found no difference after a change of coach [1, 2, 4].

## Results

### Match results

The goal count in an average soccer game (across leagues and countries) is an order of magnitude lower than in other major team sports (American football/rugby, baseball/cricket, basketball). Soccer outcomes are more subject to randomness than outcomes in other sports. Our team was no exception: in the 47 observed season games, 19 games had a goal difference of just 1 in either direction, and 14 were ties; in other words, for 33 of the 47 games (70%), a single goal would have changed the result. Goals are widely acknowledged as fickle events, often involving deflections, lapses and luck. This extent of ‘noise’ requires long sequences of games to obtain conclusive results.

Nonetheless, we found (Table 3) a significant improvement in performance after the coach changed: the average goal difference per game under the old coach (OC) was -0.24, and under the new coach (NC) +0.53. This was essentially accomplished by scoring as many goals per game while reducing the goals conceded from 1.47 to 1. Other performance measures (points, win and loss proportions) were not statistically significant but all point in the direction of performance improvement. The P-values reported in all tables are for t-tests for the mean difference between the OC regime and NC regime.

**Table 3:** Game outcomes under old coach and new coach.

	Points	Goal difference	Goals for	Goals against	Win proportion	Loss proportion
All matches	1.45	0.26	1.43	1.17	0.38	0.32
**Mean: Old coach (n = 17)**	**1.18**	**-0.24**	**1.24**	**1.47**	**0.29**	**0.41**
**Mean: New coach (n = 30)**	**1.60**	**0.53**	**1.53**	**1.00**	**0.43**	**0.27**
Difference in means: **old-new**	-0.42	-0.77	-0.30	0.47	-0.14	0.15
*(Std*. *error)*	*(0*.*39)*	*(0*.*54)*	*(0*.*44)*	*(0*.*33)*	*(0*.*14)*	*(0*.*14)*
**P-value** **H**_**a**_**: New > Old)**	0.14	**0.08**	0.25	0.92	0.17	0.85
**P-value** **H**_**a**_**: New < Old)**	0.86	0.92	0.75	**0.08**	0.83	0.15

Note: The table reports t-tests for difference in mean outcomes: old coach–new.

**Fig 2:**
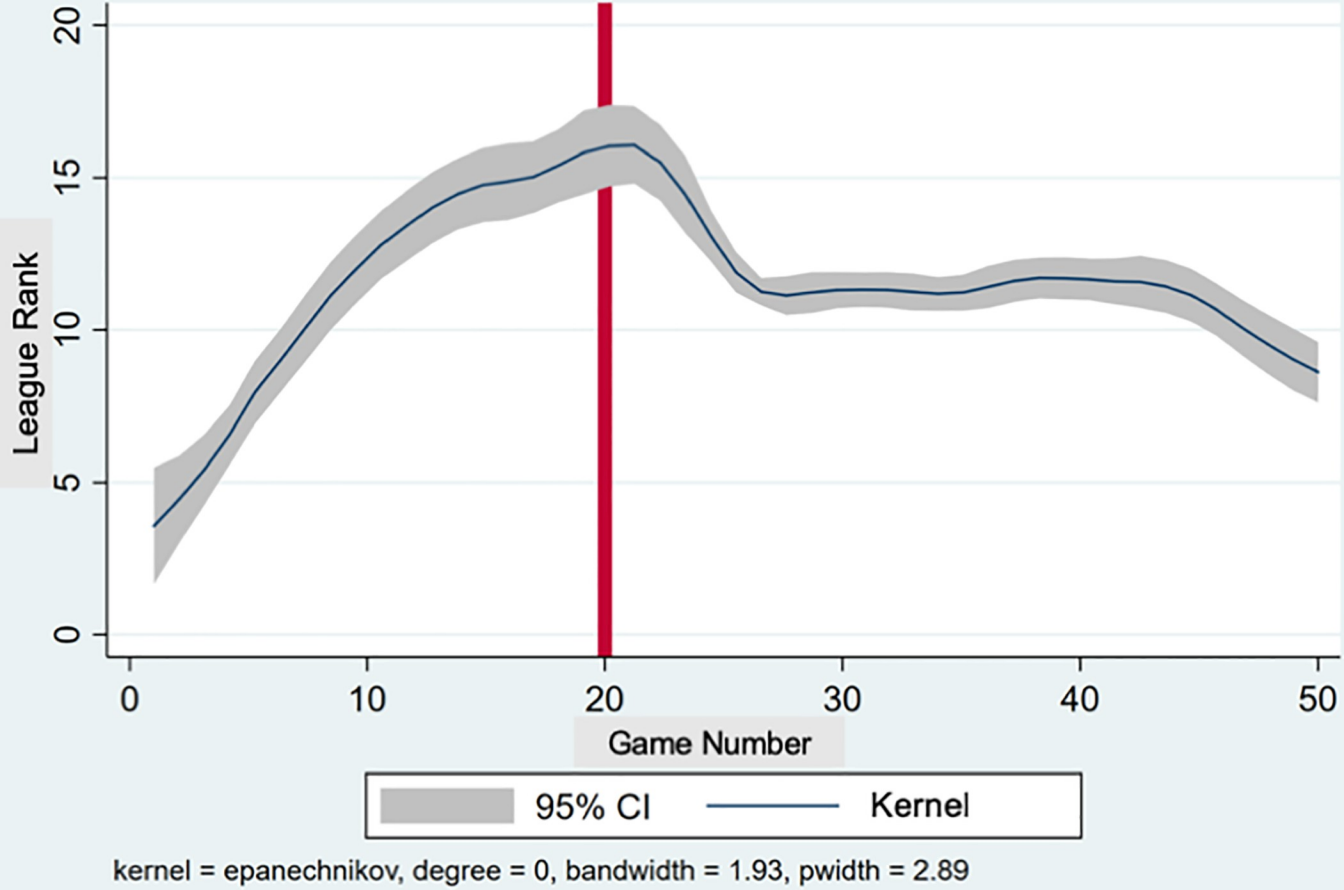
Evolution of league table position over the season (higher number represents worse rank). Note: Kernel (regression line) is a weighted local polynomial smooth regression. CI = confidence interval.

The trajectory of the league table position in Fig 2 fleshes out the performance comparison. It is based on a kernel-weighted local mean smoothed regression of the league table position of the team over the season. The kernel used is the standard epanechnikov kernel. Confidence bands (95%) are also provided. The trajectory of the league rank was *positive* under the OC (i.e., the league rank *number* increased, a *negative performance trend*, as the highest rank, No. 1, is the lowest number).

The team won the first game of the season decisively, resulting in the first rank, after which the rank deteriorated. Shortly after the NC took over, the rank reversed sharply. It stayed steady for a number of weeks before improving again. The slopes of league rank evolution reverse signs before and after the coach change at 1 per cent statistical significance. The null hypothesis that there is no structural break at match number 20, at which the new coach took over, is rejected by a breakpoint test at 1 per cent level of significance (F-statistic: 59.16, P-value ~ 0). This Chow test is based on partitioning the data into two subsamples relating to OC and NC, and comparing the restricted (single equation for the entire sample) and unrestricted (separate equations for each subsample) sum of squared residuals. The exact point of (possibly multiple) unknown structural breakpoints is determined using the Bai-Perron test which extends the approach of performing the breakpoint test at every observation over the date range. This test determined the first break-point at match number 21, one period after the new coach took over, and a second breakpoint at match number 38.

To summarize, there is support for Proposition 0 (a performance improvement after the change of coach), which does not necessarily establish that leadership succession matters (versus a ‘regression to the mean’). We now show that the performance improvement was in fact associated with systematic changes in leader behavior, and in team affective state and player behavior during games.

### Behavior of the coaches

Table 4 summarizes the counts of motivational comments made by the coach per training day, as classified into these categories. The comments were tabulated for training sessions preceding 17 games under the old coach (OC) and 25 games under the new coach (NC).

**Table 4:** Differences in coaching behaviors (week before a match) of the old and new coach.

	Praise		Support		Friendly		Challenge		Criticism	
	mild	strong	mild	strong	mild	strong	positive	negative	mild	strong
All matches (42 obs)	6.90	5.19	1.24	1.76	0.69	0.55	3.31	2.40	0.64	0.45
**Under Old coach (17 obs)**	**4.76**	**3.18**	**1.53**	**1.29**	**0.82**	**0.18**	**2.82**	**4.29**	**1.18**	**1.00**
**Under New Coach (25 obs)**	**8.36**	**6.56**	**1.04**	**2.08**	**0.60**	**0.80**	**3.64**	**1.12**	**0.28**	**0.08**
Difference in means (**old-new**)	-3.60	-3.38	0.49	-0.79	0.22	-0.62	-0.82	3.17	0.90	0.92
*Std*. *error*	*1*.*72*	*1*.*37*	*0*.*47*	*0*.*56*	*0*.*29*	*0*.*36*	*0*.*85*	*0*.*78*	*0*.*36*	*0*.*38*
**P-value (Ha: New > Old)**	**0.02**	**0.01**	0.85	**0.08**	0.78	**0.05**	0.17	1.00	0.99	0.99
**P-value (Ha: New < Old)**	0.98	0.99	0.15	0.92	0.22	0.95	0.83	**0.00**	**0.01**	**0.01**

Note: The table reports t-tests for difference in mean outcomes: old coach–new.

The NC placed significantly more emphasis on praise and support, while the OC placed more emphasis on criticism. Representative excerpts from our notes illustrate the difference in language used by the two head coaches, enriching the statistical comparisons.

September 24: At training, the energy was high and they were all running around pretty fast. The OC picked out player A and told him that all his fancy footwork was making him look like he was playing like a girl. He repeated this five or six times. The OC pointed his finger at A, close to him, and stood to the side of him and put his face very close to A’s face. A didn’t think it was funny. Later, after practice, the OC tried to make it a lighter statement and ease tension by saying it again alongside a compliment, but A still didn’t look impressed, not smiling and not giving eye contact, saying ‘I’ve got more important things to worry about than looking’.

October 1 (after a lost game): It wasn’t difficult to see how the OC was feeling after the match. Disappointed and irritated are the best words to describe it. There wasn’t a whole lot of criticism. The same rhetoric, they need to ‘start’, that they are much better than how they played, if they don’t want to play they should let him know.

October 8: The OC joined the defense practice. He seemed calm and very approachable. He got in line with the players and started moving with them in the exercise, playing along. He was very calm. Then it took a negative turn. The OC starts saying how he’s not happy with the training. ‘Hopeless. We are going to have to start filming training because you just don’t see how poorly you’re playing.’ He tells one player (who makes lots of praise of the team during this session) that he’s wrong. He asks them if they don’t want to play. ‘You want me to come in after 10 minutes and say well done? Now is the time to enjoy and play because you love the game, not because you’re getting paid.’ He is frustrated now, and it seemingly came out of nowhere; he had made no particularly negative comments leading up to this.

This illustrates that the OC was quick to criticize the players, and the strength of the challenge was somewhat unpredictable; even when things were going well, he would ‘bring them back [down] to the ground’, including his staff. This was in contrast with the new coach (NC), who from day one made an effort to be positive and supportive in his interactions with the players.

November 20 (after the first loss): The NC uses stress as a way to motivate the team, but uses it sparingly and in a very controlled fashion. This morning the players got their first proper reprimand from him. They had been late in and making last-minute phone calls to report their injuries and their performance in the morning, particularly low energy and poor mood. The NC went from being fairly quiet to quickly shouting at them, which surprised everyone and increased stress dramatically. This turned into a frantic energy, which within a few minutes had eased into increased intensity and the rest of the session was much more energetic. But this is in contrast to the OC, who would use the same technique but so often and unpredictably that the players would stay at the frantic energy level for the entire session without it calming, and after a while this seemed to become exhausting.

December 1 (after the first two wins in a row so far this season): Today is the happiest I’ve seen them this season. Two good wins in a row and some confidence and a lack of anxiety. They started with a warm-up with X in a big circle and running in a line. Warming up like this makes them look and seem much more cohesive and confident. The NC calls them into a group and with a smile on his face talks to them in a proud way, assuming they’re happy. He told them that today was gonna be fun but hard. He brought a trophy out for them to play for.

December 18 (after another win and a tie, and just before the last game before Christmas): Today was more relaxed than yesterday, or at least had less bad tension. It was just a natural step in the week, as the game day was getting closer and the pressure is on. But today they had more energy or had gotten over that bout of jitters and everyone was pretty happy. The NC had a meeting with them afterwards to talk through the detail of the opposition. He also gave them heartfelt compliments about how he thinks they are bonding more as a team now.

The statistical comparison and the illustrative quotes show that, apart from any tactical differences, the old and new coaches adopted very different styles in how they communicated with the players. This supports Proposition 1.

### Team affective tone

Table 5 shows that the team affective tone changed significantly after the change in coach. Stress levels decreased significantly (by 2 points out of 10) on average per session, and aggressiveness, competitiveness and session intensity increased by similar magnitudes.

**Table 5:** Change of team’s affective tone before and after arrival of new coach.

	Relaxed	Laughing	Stress	Ten-sion	Aggress-ion	Competi-tiveness	Cohe-sion	Intens-ity	Focussed
All (103 obs)	6.80	6.33	1.17	3.75	4.74	6.24	8.41	7.16	8.48
**Under Old coach (43 obs)**	**6.40**	**5.91**	**2.21**	**4.35**	**3.40**	**4.60**	**7.91**	**6.05**	**8.00**
**Under New Coach (60 obs)**	**7.08**	**6.63**	**0.43**	**3.32**	**5.70**	**7.42**	**8.77**	**7.95**	**8.80**
Difference in means (**old-new**)	-0.69	-0.73	1.78	1.03	-2.30	-2.81	-0.86	-1.90	-0.80
*Std*. *error*	*0*.*34*	*0*.*36*	*0*.*29*	*0*.*33*	*0*.*35*	*0*.*33*	*0*.*24*	*0*.*33*	*0*.*23*
**P-value (Ha: New > Old)**	**0.023**	**0.022**	1.000	0.999	**0.000**	**0.000**	**0.000**	**0.000**	**0.000**
**P-value (Ha: New < Old)**	0.977	0.978	**0.000**	**0.001**	1.000	1.000	1.000	1.000	1.000

Note: The table reports t-tests for difference in mean outcomes: old coach–new.

Under the NC, the players experienced fewer negative emotions in training; they put more physical energy into their attack and defense movements; and acted more to win than to merely complete the exercises, resulting in training sessions that were higher in energy and intensity. The differences indicated by the numbers reflect profound differences in the way the training sessions felt.

**Fig 3:**
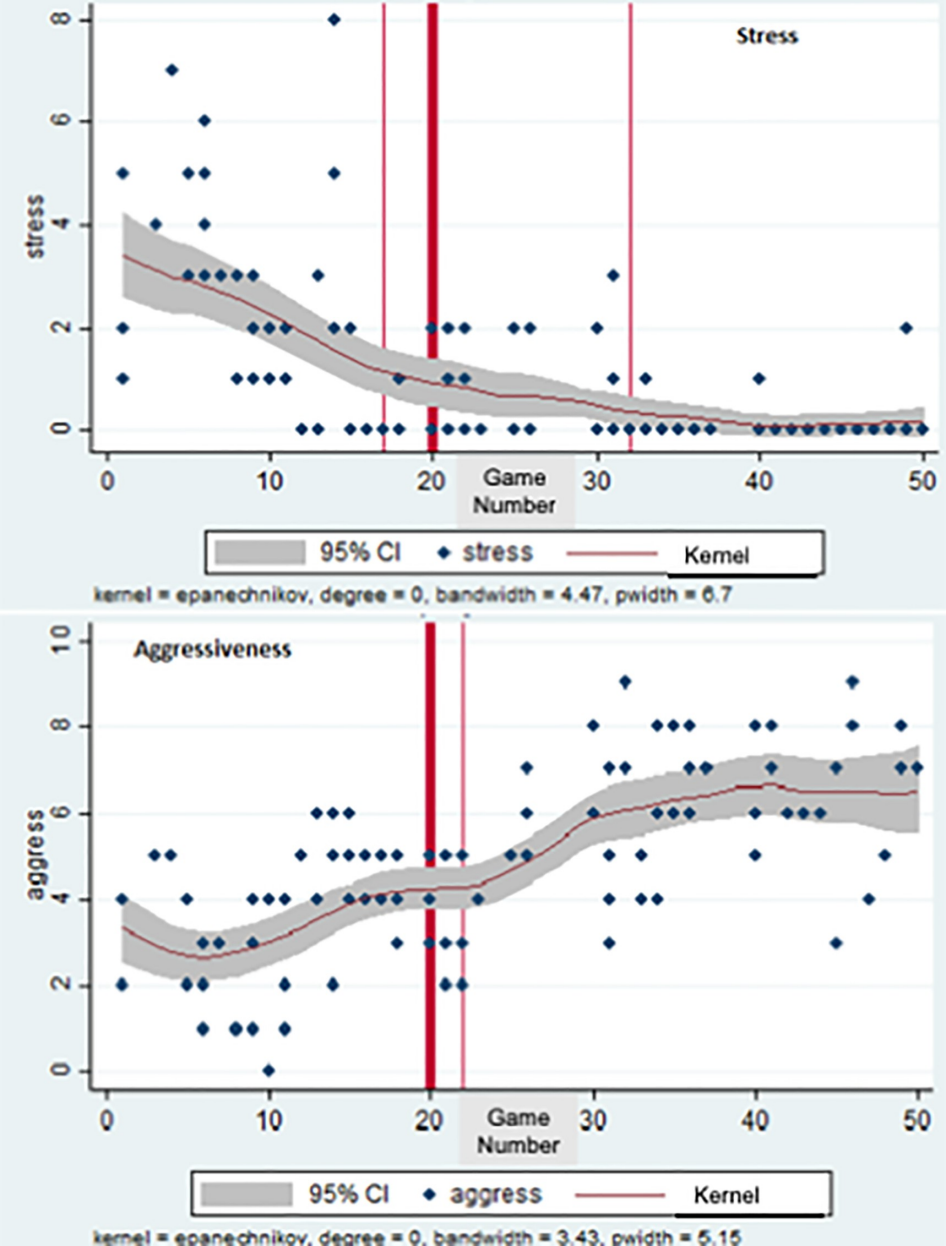
Evolution of team affective tone variables: ‘Stress’ and aggressiveness. Notes: Kernel (regression line) is a weighted local polynomial smooth regression. CI = confidence interval. The thick vertical line indicates the date of the OC leaving. The thin lines indicate structural breaks.

This is further illustrated by Fig 3, which presents the smoothed non-parametric regression of stress ratings and aggressiveness in training sessions over the season, with 95% confidence bands. Stress levels fell and grew less variable after the NC took over. There was a drop over the three weeks just prior to the OC leaving– this was actually a case of a pronounced peak in the stress (at level 8) being relieved. The peak corresponded to the following events in one training session (from the day’s notes).

The AC began the training session and was doing a good job of boosting players’ energy through aggressive encouragement and a positive attitude. The OC sat watching the team quietly for a while, then got up, walked to the pitch and abruptly told them that he expected more from them, ‘If it wasn’t for the last 1 and a half minutes today it would have a been a super running session. Somewhere along the line you have to wake up. It's embarrassing. Get to work.’ This came as a shock, and I felt the anxiety increase in the players and observed a decrease in energy and motivation.

This continued for the next half hour and I could tell the other coaches were feeling the tension based on the frustrated body language. They would occasionally put in a quick, ‘Good lads, that’s a good goal!’ in-between frustrated critique from the OC.

The OC continued to add comments about the tactics and fitness coaches at the side of the pitch, about how unhappy he was with training and some random things players were doing, such as their hands in their bibs, or one player’s hand and arm gestures. One of the players rolled his eyes when the OC started talking, which is not something you see from the players often.

The OC then spent about 30 minutes talking to the center backs about the change in tactics he wants them to do. One of them disagreed, but the OC was a good teacher and kept trying to explain what he wanted in different ways; however, it was in a loud voice with lots of swearing, almost sounding annoyed but not quite. He then took them off the pitch to have a further private conversation with them about it. The two defenders both stood with the same body language, up straight with arms on hips and chests facing the OC but taking steps backward away from him.

Toward the end of practice the energy and tension were up, but so was stress. Competitiveness and aggression were almost non-existent, and everyone was too focused on trying to play how the OC wanted them to play. Toward the end the OC calmed and the shouting stopped and competition increased a little. The day ended somewhat calmly. I don’t know what the players must think after today. Tensions were clearly high after the last match [which was lost].

After this stressful episode, there was a common feeling that this could not go on, that a change was ‘in the air’. For the following two weeks, stress levels went down (see Fig 3) as the OC calmed down, and the players readied themselves for a change. Indeed, the Bai-Perron test for multiple break-points in the stress variable finds the first break-point at match number 17, three weeks before the OC left. There was a second breakpoint at match number 32 when stress level settled to a low level with little further variability. For competitive aggressiveness, which increased under the NC, the test determined the break-point as occurring at match sequence number 22, two periods after the NC took over.

The reported excerpt from the field notes illustrates how the coach’s behavior directly impacted the affective tone of the team. This suggests that the associations in Table 5 are causal and not merely coincidental, supporting Proposition 2.

### Behaviors of players during games

We turn to the question of whether the change in the team’s affective tone mattered in terms of player behavior during games. We start with the task work, the *tactical* and athletic performance variables (distance run and sprints) that are analyzed in much of the sports performance literature. The movement variables did *not* change significantly after the change of coach. Game tactics did not change either: the passing network did not change sufficiently to be judged by experts as a tactical change (see Figure A in S1 Appendix, which compares the two passing networks); nor did the percentage of accurate passes. In summary, the athletic and tactical behavior of the players did not change, and therefore P3a is not supported. This conclusion was consistent with informal discussions with senior staff who did not see the NC as fundamentally different tactically from the OC.

However, the NC did make two significant changes. First, Table 6 shows the change in average talent. This analysis suggests that the loaned players brought in by the NC added talent to the team. Recruitment and deployment of talent is, of course, one aspect of a leader’s activity, and here it made a difference.

**Table 6:** Average player talent ratings before and after coach change.

	Average Player Talent
All (625 observations)	5.94
**Under OC (235 obs)**	**5.25**
**Under NC (390 obs)**	**6.35**
Difference in means (**OC-NC**)	-1.10
*standard error*	*0*.*14*
**P-value (Ha: NC > OC)**	**0.00**

Note: One observation is a talent rating of one player fielded in one game.

Second, the team’s affect during games, shown in Table 7, also significantly improved under the NC. Specifically, expressions of positive affect increased from 5.8 to 6.6, while expressions of negative affect remained the same.

**Table 7:** Team affect during games.

	Team Emotional Energy	
	Positive	Negative
All (40 observations)	6.33	4.70
**Mean: OC (15 obs.)**	**5.80**	**5.00**
**Mean: NC (25 obs.)**	**6.64**	**4.52**
Difference in means **(OC-NC)**	-0.84	0.48
*Std*. *error*	*0*.*67*	*0*.*70*
**P-value (Ha: NC > OC)**	**0.10**	0.76

Note: One observation is an emotional energy rating of the team over one game.

This does not provide evidence of performance relevance in itself. However, some of the qualitative observations provide additional (indirect) evidence of a performance effect. Several weeks into the season, a particularly stressful event occurred during training:

The OC stopped the training session that was being held by the AC and really laid into the team. He was disappointed by their performance. This came as a shock to the system, as the AC was very upbeat and energetic and there was a good flow going. But every few minutes when the AC boosted them up again the OC still wasn't happy and brought them back down. It was very stressful to watch, and I could tell both players and staff were tense from their body language, which ranged from stiffened (hunched shoulders and folded arms) to aggressive (hands on hips, wide stance, and body facing directly at the coach). The OC did then ease the tension but it was a significant day in the season so far.

The state of mind in the training sessions was carried over into the psychological state of the group during the match that followed this week of training:

As the game started off, they seemed totally pumped with aggressive energy, which can be beneficial but needs to be controlled, or else anxiety takes over and the energy becomes fearful and frantic. This energy continued to build–the first half of the match was intense, and as the halftime whistle blew, player Z responded to a threat from the opposition with his fist and a fight broke out. He was then sent off with a red card and the team went down to 10 men. They then conceded 3 goals and lost 3–1.

In contrast to the unpredictable mood of the OC, the NC created a steady environment through consistent behavior and the recruitment of several players who were emotionally supportive. The team state of mind appeared different shortly after the NC took over:

There had been a meeting that morning where the NC had opened the floor for the players to talk about any fears or concerns they had. What came out of the meeting was that the team were aware that they wanted to be able to be less afraid when they were playing. This was something very new that they did not do with the OC. For the rest of the session the NC’s teaching is calm. The atmosphere has a steadiness to it that it didn't before as the coach’s behavior is more predictable. The NC encouraged them to be vocal and maintained an atmosphere that never got particularly negative despite the fact they had lost the last game. (…) Competition is pretty high right from the start and there is a lot of encouragement offered from the coaches. Today seems like the optimal mood: good mood, feels calm, very competitive, seem to be especially cohesive. The group is much smaller now than it was as only the first team is here today.

The [following] game was one of the best energies I've seen them play. It was incredibly positive with nearly no negative tension. They were very aggressive and competitive and the energy they put in seemed unusually high. They clearly were very motivated to win. And they did, 3–1.

Causality is an issue in any study of this type–rather than the coach’s behavior leading to higher performance, did the coach and players behave differently because they were winning? One can address this concern of reverse causality by examining the ability of the team’s performance status (league rank) to predict the team’s affect in the games, both positive and negative. We tested three variables with respect to their effect on one another–positive team affect, negative team affect and league rank. After ensuring these variables were stationary, we regressed each one on lagged values of itself and the other two, and tested each block for significant predictive ability using F-tests. The hypothesis that league rank predicts future values of team affect score is rejected for both negative (P = 0.99) and positive affect (P = 0.41). Thus, we can conclude that team affect during games was not the result of winning or losing streaks under the OC or NC.

With the combined evidence, we conclude that the behavior of the players on the field during games changed after the change of coach in significant and performance-relevant ways that cannot be explained in terms of tactical changes. This provides support for Proposition 3b.

## Discussion and conclusion

We examined the effects of leadership succession on team performance. The empirical context is a professional soccer team, whose competitive performance was measured through the outcomes of weekly league games. Many studies have claimed, in both the management and the sports arenas, that leadership changes are inconsequential for performance. However, averaging across teams masks the importance of the leader because some new leaders may improve performance, while some do the converse. Therefore, the question of leadership succession must be studied at the detailed level of team processes. Indeed, qualitative studies have argued in favor of the importance of leaders for performance.

This paper is an analytical case study of a natural experiment in which a professional soccer team was exposed to two different coaching regimes due the abrupt change of coach mid way through the season. The context enabled us to trace out the differences between two coaching regimes in rich detail and relate it to the observed change in team performance. Our evidence suggests that the change in leadership was directly associated with changes in team processes: leader behavior, the team’s affective tone and player behavior during matches all changed, along with a change in performance. Drawing on detailed observations of all these types of change, we identified how the new coach was able to generate performance change: the pathway starts with the coach’s leadership behavior, and works through the psychology of the team, to positive affect and performance-relevant behavior during games. One strength of our study is the level of detail in the observations, which, encompassing both quantitative (longitudinal) and qualitative data, permits us to suggest causal conclusions.

Established leadership theory indicates how succession can lead to a performance change. Coaches can influence the team’s performance by simply delivering better ‘task work’, such as getting the players to be fitter, and devising better tactics. In our case, task work was unable to explain the performance increase, as the players neither ran/sprinted more, nor exhibited any significant change in passing patterns. In other words, the evidence suggests that in our case study, the coaches were comparable in task work expertise.

However, the coach can also contribute to improved performance by engaging in ‘motivational work’ through positive behavior. Our study demonstrates how a coach may lose the reins on team performance by ‘losing the team’s motivation’, while the successor of equal ‘expertise’ can harness and boost performance by investing in better team processes. Our study underscores the need for more closely observed studies of leadership changes.

An important limitation of our study comes from the fact that the detailed access came with restrictions that prevented interviews, questionnaires or physiological measurements, and allowed only one researcher on-site. Our study takes the first step in identifying performance-relevant variables in one team, while future work is needed to compare across teams to examine how the *combination* of the motivational and task work of the successor coach affects performance.

The identification of both task work (fitness and tactics) and motivational work (team affective tone during training and affect during games) points to how a successor leader may alter performance. The findings may be transferrable to management practice and offer suggestions for both coaches and management regarding how to think about the performance effects of their own behavior.

## Supporting information

S1 Appendix Descriptive statistics, correlations of variables and Figure A (passing frequencies). (DOCX)

S1 Data File Dependent and independent variable values that allow reproducing statistical results. (XLSX)
