# Supplementary material for: Management succession and success in a professional soccer team
Source: PLoS One. 2019 Mar 13;14(3):e0212634. doi: 10.1371/journal.pone.0212634 (PMC6415802; doi:10.1371/journal.pone.0212634)
Supplement: S1 Appendix — (DOCX) [file pone.0212634.s001.docx]

**S1 Appendix**

**Table A:** Descriptive Statistics of Coaching Behavior Variables


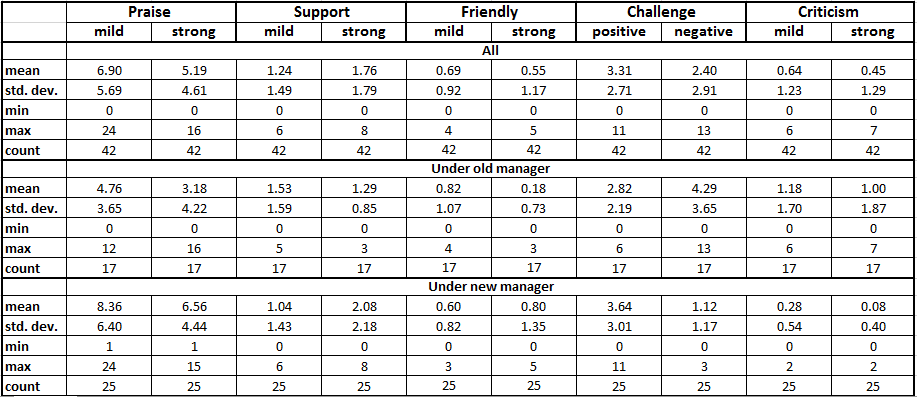


**Table B:** Correlations Among Coaching Behavior Variables


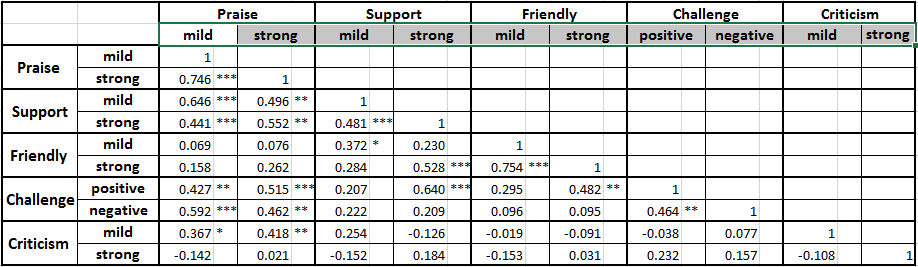


Note: *** denotes statistical significance at 1% level.

**Table C:** Descriptive Statistics of Team State-of-Mind Variables


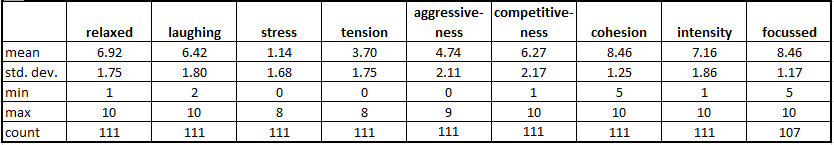


**Table D:** Correlations Among Team State-of-Mind Variables


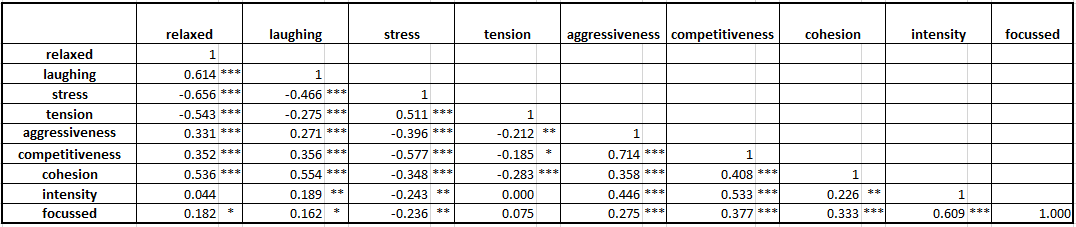


Note: *** denotes statistical significance at 1% level.

**Table E:** Descriptive Statistics of Player Talent and Team Emotional Energy


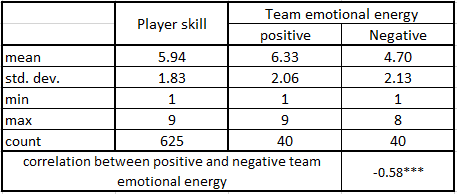


Note: *** denotes statistical significance at 1% level.

Average Passing Network Under the OC Average Passing Network Under the NC

Note: Thickness of lines represents average number of passes per game. GK = goalkeeper, (L-C-R) D = (left-center-right) defender, (L-C-M) M = (left-center-right) midfielder, (L-C-R) F = (left-center-right) forward.

**Figure A:** Comparison of Passing Networks Under the OC and the NC
